# Invasive Fungal Infections in Under-Five Diarrheal Children: Experience from an Urban Diarrheal Disease Hospital

**DOI:** 10.3390/life12010094

**Published:** 2022-01-10

**Authors:** Nusrat Jahan Shaly, Mohammed Moshtaq Pervez, Sayeeda Huq, Dilruba Ahmed, Chowdhury Rafiqul Ahsan, Monira Sarmin, Farzana Afroze, Sharika Nuzhat, Mohammod Jobayer Chisti, Tahmeed Ahmed

**Affiliations:** 1 International Centre for Diarrhoeal Disease Research, Bangladesh (icddr,b), Mohakhali, Dhaka 1212, Bangladesh; nusrat.jahan@icddrb.org (N.J.S.); pervez@icddrb.org (M.M.P.); sayeeda@icddrb.org (S.H.); dahmed@icddrb.org (D.A.); drmonira@icddrb.org (M.S.); farzanaafroz@icddrb.org (F.A.); sharika.nuzhat@icddrb.org (S.N.); tahmeed@icddrb.org (T.A.); 2 Department of Microbiology, University of Dhaka, Dhaka 1000, Bangladesh; crahsan@du.ac.bd

**Keywords:** invasive fungal infections, malnutrition, under-5-year-old children, diarrhea, death

## Abstract

Invasive fungal infections (IFIs) are opportunistic, especially in immunocompromised and hospitalized patients. Children with IFIs are more vulnerable to a fatal outcome. For early diagnosis and treatment, knowledge of the spectrum and frequency of IFIs among children is prerequisite. In this prospective observational study, we enrolled 168 children of 2–59 months old of either sex from March 2018 to December 2019 admitted to the Dhaka hospital, icddr,b. Study participants with suspected IFIs were with or without severe acute malnutrition (SAM) along with sepsis/pneumonia and fulfilled any of the following criteria: (i) failure to respond to injectable antibiotics, (ii) development of a late-onset hospital-acquired infection, (iii) needed ICU care for >7 days, (iv) took steroids/antibiotics for >2 weeks before hospitalization, and (v) developed thrush after taking injectable antibiotics. The comparison group included non-SAM (weight-for-length Z score ≥ −2) children with diarrhea and fever <3 days in the absence of co-morbidity. We performed real-time PCR, ELISA, and blood culture for the detection of fungal pathogen. Study group children with SAM, positive ELISA and PCR considered to have a IFIs. In the study group, 15/138 (10.87%) children had IFIs. Among IFIs, invasive candidiasis, aspergillosis, histoplasmosis detected in 6 (4.53%), 11 (7.97%), and 1 (0.72%) children, respectively, and (3/15 [2.17%]) children had both candidiasis and aspergillosis. Children with IFIs more often encountered septic shock (26.7% vs. 4.9%; *p* = 0.013) and had a higher death rate (46.7% vs. 8.9%; *p* < 0.001) than those without IFIs. IFIs were independently associated with female sex (OR = 3.48; 95% CI = 1.05, 11.55; *p* = 0.042) after adjusting for potential confounders. Our findings thus implicate that, malnourished children with septic shock require targeted screening for the early diagnosis and prompt management of IFIs that may help to reduce IFIs related deaths.

## 1. Introduction

Invasive fungal infections (IFIs) are foremost causes of morbidity and mortality among the critically ill and immunosuppressed pediatric patients in intensive care units (ICU) [1]. IFIs are most commonly caused by *Candida* spp., *Aspergillus* spp., *Cryptococcus* spp., *Histoplasma capsulatum*, *Coccidioide simmitis*, *Paracoccidioides* spp., and *Fusarium* spp. [2]. A study among children with acute lymphoblastic leukemia (ALL) found that the prevalence of IFI was 9.7% [3]. IFIs result from the defects in immune mechanisms among the immunosuppressed individuals and invasive procedures [1]. The pathogen enters through the puncture sites of the skin, gastrointestinal tract lesion, indwelling catheters and is spread by the hematogenous route in different parts of the body [4]. Immunodeficient children with malignancy, malnutrition, and prematurity were reported to be susceptible to develop IFIs [5]. Prolonged use of antibiotics, corticosteroids, chemotherapy, invasive procedure, and longer duration of hospital stay were identified as the critical risk factors for IFIs [6]. It was documented that IFIs were associated with higher mortality among children ranging from 23% to 48.2% [7,8].

Candidemia is the leading IFI; with an incidence rate between 4.3 to 8.1 cases/10,000 admissions [9,10]. Among IFIs, *Candida* species were found to be responsible for 59.1% of infections among pediatric acute myeloid leukemia patients in Taiwan [11]. In the gastrointestinal tract, commensal candida translocate, spread hematogenously, and colonize, resulting in deep-seated candidiasis [12]. Globally the incidence of nosocomial candidiasis has increased in infants and children in tertiary care centers. A Turkish study found that the incidence of nosocomial candidemia ranged from 3.2 to 6.9 per 1000 admissions over nine years [13]. In ICU settings, it was responsible for 10% to 42% of all bloodstream infections (BSIs) and the fourth most common BSI pathogen in children [14,15].

Among molds, *Aspergillus* spp. was the primary cause of IFI. The rate of invasive aspergillosis was around 0.4% among the hospitalized immunocompromised children in the United States [16]. In children with invasive aspergillosis, lungs and sinuses are the main sites of infection. About 85% of lung infections were found in children with probable IFD caused by *Aspergillus* [7]. However, many children may not present with the manifestation of respiratory disease. Sometimes cough, hypoxemia, and tachypnea are present in children with invasive aspergillosis [17]. In pediatric patients, the mortality rate was 15.8% and 18% with invasive candidiasis (IC) and invasive aspergillosis (IA), respectively. Both IC and IA have been reported to increase the duration of hospital stay [9,16].

Histoplasmosis is usually a self-limiting condition in children with a normal immune system. Besides the living environment, malnutrition was identified as the most important risk factor for severe and disseminated histoplasmosis due to a poor immune system. Children may present with fever, cough, headache, and lymphadenopathy. Pulmonary infiltrate was the most common finding (83%) [18].

A study done by Gurgani and his colleagues revealed the burden of several serious fungal infections in Bangladesh (28). Superficial mycoses and dermatomycoses are common in rural areas and tertiary level hospital outpatient departments [19,20]. Systemic mycoses are also documented as a significant emerging problem [21,22,23,24,25,26]. In developing countries like Bangladesh, the high malnutrition rates [27] with accompanying immunosuppression may result in a higher burden of IFIs. Additionally, immunosuppressed kids are at risk of developing dispersed candidiasis [28]. However, life-threatening invasive fungal infections are presumed to be increasing in Bangladesh. Data on the prevalence of invasive fungal infections among critically ill or ICU admitted children are limited in Bangladesh [29]. Moreover, diagnosis of IFI is difficult. However, fungal culture and microscopy are considered the gold standard with some limitations. The newer tests Platellia^TM^ Aspergillus for galactomannan antigen, Fungitell kit for beta-D-Glucan detection, or PCR assay are either unavailable or not performed routinely in most healthcare settings in Bangladesh [30]. Hence, we observed increasing use of systemic anti-fungal for critically ill or ICU patients who were non-responsive to front-line antibiotics. Thus, patients may suffer from drug related side effects or risk of emergence of drug-resistant fungal strains [31,32].

A study conducted in the Dhaka hospital of icddr,b among under-5-year-old children with diarrhea and bacteremia from Dhaka hospital found that only 17% had bacterial isolates from blood, and 10% were pathogenic bacteria. Comparing death and survivor, bacterial isolates in stool were 25% and 26%, respectively [33]. It was perceived that IFIs may be prevalent in these children. However, there is inadequate information regarding the proportion, presenting features, and outcome of these hospitalized children having invasive fungal infections. To develop an optimal management protocol, we require extensive understanding of the epidemiology of IFIs. Thus, our study was focused on determining the spectrum and frequency of invasive fungal infections caused by *Candida* species, *Pneumocystis jirovecii*, *Aspergillus* species, *Cryptococcus* species, *Histoplasma Capsulatum*, and their associated factors, outcome among under-5-year-old diarrheal children.

## 2. Materials and Methods

### 2.1. Ethics Statement

This study was approved (protocol number-PR-17069) by the institutional review boards (IRB) of the International Centre for Diarrheal Disease Research, Bangladesh (icddr,b), comprised of the Research Review Committee (RRC) and the Ethical Review Committee (ERC). Signed informed consents were obtained from the parents/legal guardians of the participating children before enrollment. The consent form was written and formatted in Bengali for a proper understanding of the caregivers.

### 2.2. Study Setting

The study was conducted at the Dhaka Hospital of icddr,b between March 2018 and December 2019. Critically ill patients were treated in the Intensive Care Unit (ICU) of Dhaka Hospital, equipped with necessary life support measures, including non-invasive and invasive ventilator supports. According to the 2019–2020 annual report, Dhaka hospital, the largest diarrheal treatment center in the world, treated around 200,000 patients. More than half of these patients (57.2%) were under five years of age. Among under-5-year-old children, those with severe acute malnutrition (SAM) with or without severe pneumonia and/or sepsis conventionally receive broad-spectrum antibiotics. icddr,b possesses a well-equipped diagnostic laboratory facility capable of performing all the clinical tests, including real-time PCR (Polymerase Chain Reaction), ELISA (Enzyme-Linked Immunosorbent Assay) required to detect fungal etiology.

### 2.3. Study Design

It was a prospectively conducted observational study.

### 2.4. Study Group

The study children with suspected invasive fungal infections were 2–59-month-old children of either sex with or without severe acute malnutrition (SAM) along with sepsis/pneumonia and fulfilled any of the following criteria: (i) Failed to respond injectable antibiotics (first line—Ampicillin plus gentamicin, second line—Ceftriaxone plus levofloxacin); (ii) children with SAM or had a history of recent measles or any condition that induced immune suppression plus failure to respond to first line antibiotics (first line antibiotics—Ampicillin + Gentamicin); (iii) developed a late-onset hospital-acquired infection; (iv) needed ICU care for more than seven days; (v) took steroids or antibiotics for more than two weeks before hospitalization; or (vi) developed thrush after taking second line antibiotics.

### 2.5. Comparison Group

Children from the short-stay unit (SSU) or outpatient department (OPD) of Dhaka hospital were eligible for the comparison group. They had either diarrhea or fever (less than 3 days) but non-SAM (Weight-for-length Z score ≥−2). We excluded the children with cough, persistent diarrhea, congenital anomaly, or medical/surgical co-morbidity or who received antibiotics for the current illness before hospitalization.

### 2.6. Sample Size

#### 2.6.1. Study Group

A study from the India found that the prevalence of nosocomial candidemia infection among the general population was 6.5% [34,35] and another US based study among HIV-infected children from the out-patient department was 6% [36]. We anticipated a higher prevalence of IFIs among our study children who failed to respond to antibiotics for pneumonia or sepsis. With 80% power and 5% type I error and considering 10% prevalence of IFIs among 2–59 months old children in our in-patient ward, we required 138 children.

#### 2.6.2. Comparison Group

We observed most of our study children were positive for at least one fungal pathogen with or without the potential fungal disease. A proportion of them treated with the antifungal drug depending on the adjudication of ICU consultants. To mitigate the question of whether these fungi were responsible for the IFIs among the study children or not, we selected a comparison group from healthy children. Therefore, we enrolled 30 healthy children purposively after completion of enrollment of study children by an amendment of the protocol approved by our IRB.

### 2.7. Patient Management

According to the hospital’s standard management guidelines, the enrolled children received management either in the ICU or LSU. We treated a few patients with antifungal drugs: amphotericin B, fluconazole, and voriconazole. Antifungal treatment was given based on the patient’s clinical signs, symptoms, detected fungal pathogens, and judgment of the treating consultant. Additional required managements obtained from the hospital have been described elsewhere [37,38,39].

### 2.8. Measurements

A pretested Case Record Form (CRF) was used to gather relevant information for the study. We collected clinical data included the nature and duration of diarrhea, fever, cough, and respiratory distress; medication history, especially antibiotic for current illness before hospitalization, a history of previous hospitalization other than the present illness; clinical examination data regarding pulse, respiratory rate, axillary temperature, and oxygen saturation. Socio-demographic characteristics included age, sex, parental age with education, parent’s occupation, monthly family income, and history of breastfeeding. We also measured the weight, and height of the study participants for calculation of WHO standard Z score, mid-upper arm circumference as anthropometric measurements. Laboratory test results such as lactate dehydrogenase (LDH) level >299 U/L, Beta-D-Glucan level in the serum >80 pg/mL, and optical density (OD) for Galactomannan >0.5 were considered as positive. Complete blood count (CBC) was performed at different time points considering the patient’s clinical condition. To maintain homogeneity, we included CBC reports for analysis, which were done within 72 h of enrollment, and hemoglobin levels ≤9.3 g/dL were considered as having anemia [40].

### 2.9. Definition

We followed the WHO definition of severe acute malnutrition, pneumonia, and severe pneumonia [40,41], surviving sepsis guidelines [42] and also previously conducted study from icddr,b for sepsis, severe sepsis, and septic shock in diarrheal children [37,43].

#### 2.9.1. Danger Signs of Severe Pneumonia

According to the WHO, severe pneumonia was defined if a child had hypoxemia, cyanosis, inability to breastfeed or drink, or grunting, and general danger signs including lethargy, reduced consciousness, and seizures during enrollment [40].

#### 2.9.2. Critically Ill

According to the pediatric intensive care society, children requiring the support of high dependency or intensive care unit [44] are considered critically ill.

#### 2.9.3. Late-Onset Hospital-Associated Infection (LOHAI)

LOHAI is defined as infection that appears 96 h after hospital admission in a patient but was absent or not incubating at the time of hospital admission. LOHAI also includes an infection occurring within three days of discharge from the hospital [40].

#### 2.9.4. Invasive Fungal Infection (IFI)

In December 2019, the European Organization for Research and Treatment of Cancer and the Mycoses Study Group have published a revised and updated definitions of invasive fungal disease. They categorized invasive fungal diseases (IFDs) into three groups, “proven”, “probable”, and “possible” IFD [45]. Considering our clinical context, we used a modified definition of IFD. Children with SAM and positive for Galactomannan (GM) antigen, Beta-D-glucan, and real-time PCR on a blood sample were considered as having IFIs.

### 2.10. Laboratory Investigations

We performed assay for LDH, real-time PCR and ELISA (Platellia^TM^ Aspergillus Ag for the detection of galactomannan, and Fungitell kit for the detection of (1→3)-β-D-glucan) and blood culture for the detection of *Candida* spp., *Histoplasma capsulatum*, *Aspergillus* spp., and *Pneumocystis jiroveci* from blood and nasopharyngeal wash; *Histoplasma capsulatum* and *Cryptococcus* spp. from urine.

### 2.11. Molecular Detection of Fungal Pathogen

A study physician collected blood and other biological specimens following the standard operating procedure (SOP) and the samples were transported to the laboratory within 30–60 min of the collection. At the laboratory, designated lab personnel extracted total nucleic acid from each specimen using QIAamp DNA mini kit (QIAGEN) and performed real-time PCR (using ABI7500 Dx fast instrument from Applied Biosystems, Foster City, CA, USA) to identify invasive fungal pathogens named, *Candida* spp., *Aspergillus* spp., *Histoplasma capsulatum*, *Pneumocystis jirovecii*, and *Cryptococcus* spp. using TaqMan probes [46,47,48]. We had used single plex TaqMan PCR with a positive and negative control in each run for the detection of fungal pathogens in all samples. We used all TaqMan PCR primer and probe from the published article and verified with beacon designer software. For identification of the fungal antigen by ELISA, we had used Fungitell and Platellia^TM^ Aspergillus Ag kit. Fungitell is an FDA approved, highly sensitive rapid diagnostic test kit that identifies (1→3)-β-D-glucan in serum within 1 h. Fungitell is a pan fungal marker used to identify various fungal pathogens like, *Candida* spp., *Aspergillus* spp., *Histoplasma capsulatum*, *Pneumocystis jirovecii*, and Platellia^TM^ Aspergillus Ag kit (from Bio-Rad) for the detection GM of *Aspergillus* species.

### 2.12. Data Analysis

Data were entered into a statistical package for the social sciences [IBM SPSS Statistics for Windows, Version 20.0. Armonk, NY, USA: IBM Corp] and Epi-Info (version 7.0, USD, Stone Mountain, GA, USA). For descriptive analysis, we had calculated frequencies, median, means, and standard deviation as appropriate to know the proportion of IFIs caused by *Candida* species, *Aspergillus* species, *Histoplasma capsulatum*, *Cryptococcus* species, and *Pneumocystis jirovecii*. Categorical variables compared using Pearson’s Chi-square test. In contrast, the Student’s t-test was used to compare the normally distributed continuous data, and Mann–Whitney U tests was used to compare non-parametric data. We considered a two-sided probability of less than 0·05 statistically significant, and the odds ratio (OR) and their 95% confidence intervals (CI) were calculated to determine the strength of association. Finally, a logistic regression analysis was done to control the confounders.

## 3. Results

**Figure 1:**
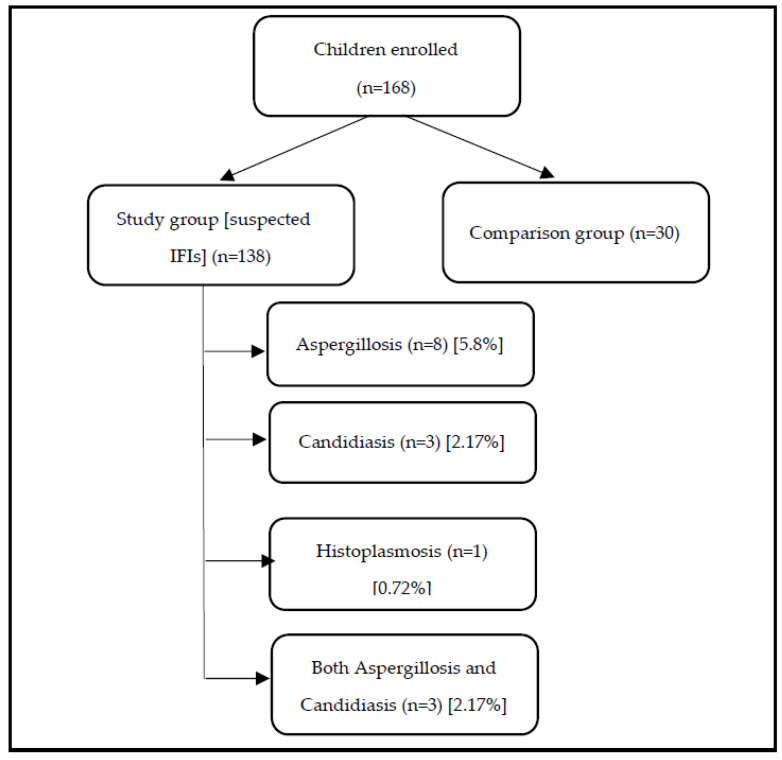
Proportion of children with invasive fungal infections (IFIs).

Among 168 enrolled under-5-year-old children, 138 were in the study group, and 30 were in the comparison group are presented in the Figure 1. In the study group, 15 children were with IFIs. In IFIs, six children were *Candida* spp. positive by PCR from the blood sample. The proportion of invasive candidiasis was 4.53% (6/138), and within the IFIs group, 40% (6/15) were found to be positive for *Candida* spp. *Aspergillus* spp. was positive by PCR from the blood of 11 children among the IFIs group. The proportion of invasive aspergillosis was 7.97% (11/138) and within the IFIs group was 73.33% (11/15). The proportion of invasive histoplasmosis by PCR on blood was 0.72% (1/138) and was 6.67% (1/15) within the IFIs group. In the IFIs group, three children had both invasive candidiasis and aspergillosis by PCR on blood was 0.72% (1/138) and was 6.67% (1/15) within the IFIs. In the IFIs group, three children had both invasive candidiasis and aspergillosis.

Table 1 shows that 36.2% (50/138) were female in the study group, whereas, in the comparison group, 20% (6/30) were female. The median age in months was 8 months in the study group and 9.5 months in the comparison group. The study group had less monthly family income (Table 1) and more under-fifth-grade paternal education than the comparison group (Table 1). Regarding maternal education, both groups were comparable (Table 1). A significant number of children (78.3%) in the study group were not exclusively breastfed, had a history of previous hospital admission and fever compared to the comparison group (Table 1). The study group had lower MUAC, higher respiratory rate, and heart rate than the comparison group. The median level (interquartile range) of LDH (in U/L) and sample optical density (OD) for galactomannan (GM) was more in the study group compared to their counterpart. The study group was more likely to have higher LDH, PCR (blood), and Aspergillus positive than the comparison group. The Median (interquartile range) duration of hospitalization (in days) was more in the study group than in the comparison group. B-D-Glucan was positive in 54.3% (75/138) of the children in the study group.

Table 2 shows that the clinical outcomes of children with IFIs were sick, and many of them (13/15 [87%]) required ICU care and (7/15 [47%]) died. Two patients (2/15 [13%]) were referred, and both of them died (also Figure S1, supplementary file).

Table 3 shows the comparative characteristics of children with and without IFIs within the study group. Children with IFIs more often had female sex than those without IFIs. The median (inter-quartile range) age (in months) was comparable between the study groups. Parent’s age, education, occupation, breastfeeding status of the children, antibiotic use history, and co-morbidity were also comparable between the study groups. Children with the IFIs compared to those without IFIs more frequently received inotrope during their hospital stay. Their death rate was significantly higher than those without IFIs. Danger sign of severe pneumonia was more in children with IFIs compared to those without IFIs.

From the Table 4, logistic regression analysis found that children with invasive fungal infections were independently associated with female sex after adjusting for potential confounder.

## 4. Discussion

Globally, immunocompromised children are more susceptible to develop IFIs [2]. According to the best of our knowledge, this was the first study in Bangladesh that evaluated the spectrum, frequency, associated factors, and outcome of IFIs among hospitalized ill children admitted in the critical care ward. The identification of around 11% probable IFIs among our study children is the most important observation. 4.53%, 7.97%, and 0.72% of the children with IFIs had invasive candidiasis, aspergillosis, and histoplasmosis, respectively. Our objective was to identify fungal pathogen (*Candida* species, *Pneumocystis jirovecii*, *Aspergillus* species, *Cryptococcus* species, and *Histoplasma Capsulatum),* and the detection of specific species was beyond our scope. *Candida* spp. were associated with suppressed cell-mediated immune response, and it was the most prevalent fungal pathogen in malnourished children [49]. In developing countries like Bangladesh, invasive aspergillosis is not rare. A study from Bangladesh conducted with the aim of identifying the histopathology and etiology of childhood pneumonia found *Pneumocystis carinii* (PC) and *Aspergillus* in 4% and 3% autopsy patients, respectively. This opportunistic infection is primarily due to malnutrition and weakened host defenses throughout persistent serious illnesses [50]. Disseminated histoplasmosis could be found in children with no underlying disease other than malnutrition [51]. In our study, we did not find any children who were positive for *Pneumocystis jirovecii* and *Cryptococcus* spp. positive. Perhaps it might be due to the low prevalence of HIV in the general population in Bangladesh [52]. A study from the HIV unit of Dhaka hospital of icddr,b showed that 24 HIV-infected children were admitted over three years. Among them, 16.7% children had *Pneumocystis jirovecii pneumonia* (PCP) [53]. Cryptococcosis and histoplasmosis are not frequent in Bangladesh. Bangladesh is still a low prevalent country for HIV. The first reported case of disseminated Histoplasmosis in Bangladesh was in 1982 [54]. A systematic review on Histoplasmosis from Bangladesh found 26 patients over 55 years from 1962 to 2017. All were male patients aged 8–75 years, and four had HIV/AIDS. Disseminated histoplasmosis was present in 22 patients, and four patients had localized oropharyngeal disease [55]. Among under-5-year-olds there was a case report of disseminated histoplasmosis in a 3-and-a-half-year-old boy from Bangladesh [56]. Our study also found a deficient proportion of invasive Histoplasmosis in under-5-year-old children. Shahrin et al., found six presumptive cryptococcal meningitis cases among HIV-infected adults over three years [57]. Based on this data, it was estimated that the rate of cryptococcal meningitis was 0.01 (15/100,000) in each year in Bangladesh among HIV infected patients [29]. From Bangladesh, we do not find patients with cryptococcal meningitis in non-HIV patients except one case report of post-renal transplant cryptococcal meningitis [26]. In our study, we did not find any children positive with cryptococcal infection.

The European Organization for Research and Treatment of Cancer and the Mycoses Study Group Education and Research Consortium (EORTC/MSGERC) published a consensus definition of invasive fungal diseases (IFDs) in December 2019. According to the classification, IFD is divided into three groups, “proven”, “probable”, and “possible”. Regarding a patient’s immune status, any patient can be categorized as proven IFD if fungus is detected by microscopy or culture of the sterile material or blood culture or tissue nucleic acid diagnosis and DNA sequencing. However, probable and possible IFD are categorized only for immunocompromised patients except for endemic mycosis. There must be three-factor to be diagnosed as a probable IFD; namely host factors, clinical features, and mycological evidence. In this revised and updated definition of 2019, EORTC considered Platellia^TM^ Aspergillus, Fungitell, and PCR as the tests for diagnosing probable IFD [45]. However, the cutoff values for these mycological tests are different from the ones that we used in our study. Since we had completed our study before this definition was published. Possible IFD defined as having host factors and clinical features without mycological evidence. Under such circumstance, we made a consensus definition of probable IFIs for our study patients. Those with severe acute malnutrition as a host factor, plus sepsis or pneumonia or both as a clinical feature, plus positive real-time PCR on blood, and Platellia^TM^ Aspergillus, and Fungitell as a mycological evidence were considered to be a probable IFD or invasive fungal infection.

The mortality rate was significantly higher in children with IFIs compared to those without IFIs. Children with IFIs had severe malnutrition whereas many of the children without IFIs did not have severe malnutrition due to purposeful categorization of probable IFIs. Malnutrition is one of the important causes of secondary immunodeficiency where innate and acquired immune systems might be impaired, resulting in increased susceptibility to gastrointestinal and respiratory infections [58,59] and often leading to deaths [60]. Moreover, children with IFIs more often presented with danger signs of severe pneumonia and experienced septic shock than those without IFIs. Previous studies in Bangladesh found that the case-fatality rate was significantly higher in children experiencing severe pneumonia [38] or septic shock [37] compared to those who did not have these entities. Studies outside Bangladesh also revealed that children who presented with septic shock at the time of candidemia had a higher death rate (71.4% vs. 12.5%) than those who presented with only fever [61]. Among non-neonatal pediatric episodes of invasive candidiasis, septic shock developed in approximately 21.8% cases, and attributable mortality risk was in 17.5% cases, and compared to treatment success (67.9% vs. 13%), failure was more in the pediatric patient who had a septic shock at the onset of invasive candidiasis [62]. Surviving sepsis guidelines for children underscored the bacterial cause of sepsis in children [42]. However, in our day-to-day practice, sepsis due to fungal infection was found to be expected in malnourished children. Sepsis due to invasive fungal infections was observed to be mainly limited to immune-compromised children. A study conducted in East Africa, among malnourished hospitalized children, found that about 45% of children had diarrhea, 12% had candidiasis, and 55% died due to sepsis, and the findings of this study are consistent with our study findings [63]. In our study, some of the reports were available after the death of a child. A few children received antifungal drugs also. However, as this was an observational study and standard care at icddr,b does not include antifungal empirically, we were unable to conclude the role of antifungal therapies in the IFI affected children.

The observation of the independent association of females with invasive fungal infection is really interesting. In countries like Bangladesh, gender bias starts from birth to throughout life. In childhood, gender bias was observed during family food allocation, education, and healthcare-seeking behavior. Female children were more likely to be severely malnourished than male children [64]. Another study from Bangladesh reported risk factors for dehydrating diarrhea and found dehydration was more common among hospitalized females than males [65]. Probably lack of healthcare-seeking behavior for a female child made them more vulnerable. A study from India conducted in a tribal community reported that mothers sought less healthcare for female children compared to male children (58% vs. 83.9%), and no treatment for illness were more evident for the females than the males (42% vs. 16.5%) [66]. A systematic review on healthcare behavior for neonates from South Asia also observed similar results and found less care-seeking behavior and higher mortality among female neonates than males [67].

Our bivariate analysis observed that the lack of exclusive breastfeeding, antibiotics use before hospitalization, danger signs of pneumonia, and anemia was higher in children with IFIs than those without IFIs. However, these variables were not found to be statistically significant in the multivariate regression analysis, probably due to our small sample size. It was well reported that breast milk protects against infections through specific and non-specific immune factors. Human milk enhances the immature immunological system among young infants and strengthens host defense mechanisms against infective and other foreign agents [68]. Moreover, anemia also aggravates this immune disturbance, especially in malnourished children [69].

Another foremost observation from our study was that fever was less common in children with IFIs than those without IFIs. As SAM was the main component of IFIs in our study, a reduced febrile response during infection is usually associated with low serum albumin and retinol-binding protein in malnutrition. Endogenous pyrogen/interleukin-1, essential for stimulating fever, is less in the amount in malnourished children [70]. Moreover, immunosuppression and lack of standard defense mechanisms in malnourished children make it hard to get classical signs of infection such as fever, pain, and inflammation [71].

Our study had several limitations. First of all, the comparison group was small compared to the study group. However, we believe the comparison group though small, added valuable background information to this study. Secondly, we were unable to perform the ELISA by Fungitell kit (to test Beta-D-Glucan) in the comparison group during the study period. Thirdly, we did not perform an HIV test for our study children as Bangladesh is low-prevalent for HIV. In our study, we had only one child with positive fungal blood culture. The isolated fungal pathogen was *Candida ciferrii*, a potential skin contaminant. It is noteworthy that the patient improved with ongoing antibiotics did not require any antifungals, and was discharged from the hospital after full recovery. We followed the child up to 6 months after discharge from the hospital. The patient was alive and not found to have significant complaints during follow-up.

## 5. Conclusions

Our study data demonstrate that the prevalence of probable IFIs in Bangladesh is 10.87% and is predominated by candidiasis and aspergillosis. Children with IFIs have a higher mortality rate than those without IFIs. Female children are found to be independently associated with IFIs. Identifying simple clinical risk factors may assist the clinicians in early diagnosis and treatment of IFIs that may further aid in reducing the morbidity and mortality in such children, especially in resource-limited settings.

## Figures and Tables

**Table 1:** Baseline characteristics of the study children.

Variables		Study Group *n* = 138 (%)	Comparison Group *n* = 30 (%)	OR (Unadjusted 95% CI)	*p* Value
Sex (Female)		50 (36.2)	6 (20)	2.27 (0.87–5.93)	0.134
Age in months (median, IQR)		8.0 (5.0, 10.0)	9.5 (8.0, 12.5)		0.001
Father’s age in years (mean ± SD)		30.78 (±6.9)	30.1 (±5.5)		0.63
Mother’s age in years mean ± SD)		24 (±5.6)	23.9 (±4.0)		0.85
Monthly income in USD (median, IQR)		179 (119, 239)	209 (179, 299)		0.038
Father’s education	No or under five grades	63 (45.7)	6 (20)		0.034
	Six to ten grades	39 (28.3)	12 (40)		
	More than ten grades	36 (26.1)	12 (40)		
Mother’s education	No or under five grades	55 (39.9)	11 (36.7)		0.537
	Six to ten grades	46 (33.3)	8 (26.7)		
	More than ten grades	37 (26.8)	11 (36.7)		
Day labor father		63 (45.7)	10 (33.3)	1.68 (0.73–3.85)	0.302
Housewife mother		127 (92)	28 (93.3)	0.82 (0.17–3.93)	1.000
Not exclusively breast feed		108 (78.3)	17 (56.7)	2.75 (1.20–6.30)	0.026
Lives in slum		11 (8.0)	5 (16.7)	0.43 (0.14–1.35)	0.259
H/o previous hospitalization		67 (48.6)	3 (10)	8.49 (2.46–29.30)	<0.001
Diarrhea		71 (51.4)	30 (100)		<0.001
Vomiting		20 (14.5)	9 (30)	0.39 (0.16–0.99)	0.076
Cough		122 (88.4)	0		<0.001
Fever		98 (71.0)	6 (20)	9.8 (3.73–25.78)	<0.001
Poor oral intake		50 (36.2)	0		<0.001
Respiratory distress		78 (56.5)	0		<0.001
SAM		72 (52.2)	0		<0.001
MUAC in cm (mean ± SD)		11.34 (±1.76)	14.66 (±0.78)		<0.001
Respiratory rate, breaths/min (mean ± SD)		50 (±12)	33 (±5)		<0.001
Heart rate, beats/min (mean ± SD)		140 (±19)	124 (±10)		<0.001
Galactomannan (GM) positive		62 (44.9)	8 (26.7)	2.24 (0.93–5.39)	0.102
Sample OD for GM (median, IQR)		0.46 (0.35, 1.56)	0.37 (0.23, 0.62)		0.002
B-D-Glucan positive		75 (54.3)	Not done		
LDH (median, IQR)		487 (364, 675)	299 (254, 351)		<0.001
LDH positive		125 (90.6)	15 (50)	9.62 (3.85–24.02)	<0.001
PCR positive on Blood		50 (36.2)	2 (6.7)	7.95 (1.82–34.80)	<0.001
*Candida* spp.		16 (11.6)	0		0.079
*Aspergillus* spp.		34 (24.6)	2 (6.7)	4.58 (1.04–20.23)	0.028
* Histoplasma * spp.		7 (5.1)	0		0.354
PCR positive on urine (Histoplasma only)		8 (5.8)	0		0.353
Duration of hospitalization in days (median, IQR)		16.0 (11.0, 24.00)	1.0 (1.0, 2.0)		<0.001

Abbreviation: SAM = Severe acute malnutrition, MUAC = Mid upper arm circumference, LDH = Lactate dehydrogenase, OD = Optical density.

**Table 2:** Characteristics and outcomes of the children having invasive fungal infections during hospitalization.

DOA	Age in Months	Sex	Hypoxemia at Enrolment	Needed ICU Care	Underlying Clinical Condition		PCR on the Blood Sample			Outcome
					Pneumonia	Sepsis/Severe Sepsis/Septic Shock	*Candida* (*n* = 6)	*Aspergillus* (*n* = 11)	*Histoplasma* (*n* = 1)	
05.03.2018	7	Female	No	yes	Yes	Yes	Positive	Positive	Negative	Dead
06.04.2018	11	Female	No	yes	Yes		Positive	Negative	Negative	Survived
02.05.2018	8	Male	No	no	Yes		Positive	Positive	Negative	Survived
11.05.2018	6	Female	No	yes		Yes	Negative	Negative	Positive	Survived
29.06.2018	12	Female	No	yes	Yes	Yes	Negative	Positive	Negative	Dead
08.08.2018	2	Female	No	yes	Yes		Positive	Positive	Negative	LAMA
16.08.2018	30	Male	Yes	yes	Yes	Yes	Negative	Positive	Negative	Referred
11.09.2018	4	Female	Yes	yes	Yes	Yes	Positive	Negative	Negative	Dead
02.10.2018	3	Male	No	yes	Yes	Yes	Negative	Positive	Negative	Dead
14.11.2018	11	Male	Yes	yes	Yes	Yes	Negative	Positive	Negative	Referred
03.01.2019	4	Female	Yes	yes	Yes	Yes	Negative	Positive	Negative	Dead
25.01.2019	10	Female	Yes	yes	Yes		Positive	Negative	Negative	Survived
21.02.2019	47	Female	Yes	yes	Yes	Yes	Negative	Positive	Negative	Dead
01.02.2019	8	Male	No	no	Yes		Negative	Positive	Negative	Survived
22.03.2019	3	Female	Yes	yes	Yes		Negative	Positive	Negative	Dead

Abbreviation: LAMA- Left against medical advice, DOA- Date of Admission.

**Table 3:** Characteristics and outcome of children with and without invasive fungal infections.

Variables		Children with IFI *n* = 15 (%)	Children without IFI *n* = 123 (%)	OR (Unadjusted 95% CI)	*p*-Value
Female sex		10 (66.7)	40 (32.5)	4.15 (1.33–12.95)	0.019
Age in months (median, IQR)		8.0 (4.0, 11.0)	8.0 (5.0, 10.0)		0.836
Father’s age in years (mean ± SD)		31.1 ± 8.2	30.7 ± 6.7		0.836
Mother’s age in years mean ± SD)		23.8 ± 6.1	24.1 ± 5.5		0.847
Father’s education	No or under five grades	8 (53.3)	55 (44.7)		0.731
	Six to ten grades	3 (20.0)	36 (29.3)		
	More than ten grades	4 (26.7)	32 (26.0)		
Mother’s education	No or under five grades	9 (60.0)	46 (37.4)		0.215
	Six to ten grades	4 (26.7)	42 (34.1)		
	More than ten grades	2 (13.3)	35 (28.5)		
Day labor Father		7 (46.7)	56 (45.5)	1.04 (0.36–3.06)	0.848
Housewife Mother		12 (80.0)	115 (93.5)	0.28 (0.06–1.19)	0.100
Monthly income in USD (median, IQR)		143 (119, 179)	179 (119, 239)		0.297
Not Exclusively Breast feed		13 (86.7)	95 (77.2)	1.92 (0.41–9.00)	0.522
Duration of breast feeding in months (median, IQR)		4.0 (1.0, 8.0)	6.0 (2.5, 9.0)		0.197
Antibiotic use before hospital admission		12 (80.0)	88 (75.2)	1.32 (0.35–4.99)	1.000
Developed thrush after hospital admission		4 (26.7)	34 (27.6)	0.95 (0.28–3.19)	1.000
H/o previous hospitalization		6 (40.0)	61 (49.6)	0.68 (0.23–2.01)	0.668
Present co-morbidity		2 (13.3)	14 (11.4)	1.19 (0.24–5.86)	0.685
Diarrhea		7 (46.7)	64 (52.0)	0.81 (0.28–2.36)	0.905
Cough		11 (73.3)	111 (90.2)	0.29 (0.08–1.08)	0.074
Fever		8 (53.3)	90 (73.2)	0.42 (0.14–1.25)	0.194
Respiratory distress		8 (53.3)	70 (56.9)	0.86 (0.29–2.54)	0.990
Poor oral intake		7 (46.7)	43 (35.0)	1.63 (0.55–4.79)	0.544
Radial pulse in minute (mean ± SD)		136.5 ± 18.0	140.5 ± 19.5		0.450
Respiratory rate in minute (mean ± SD)		48.0 ± 10.8	50.2 ± 12.1		0.505
Developed septic shock (received inotrope)		4 (26.7)	6 (4.9)	7.09 (1.24–34.75)	0.013
Lower chest wall in drawing		9 (60)	71 (57.7)	1.09 (0.37–3.27)	0.913
Danger sign of severe pneumonia		8 (53.3)	27 (22.0)	4.06 (1.16–14.31)	0.022
Crackles		10 (66.7)	91 (74)	0.70 (0.22–2.21)	0.767
Absolute neutrophil count (median, IQR)		6469.8 (2694.9, 17,796.4) Missing 7	7279.1 (4580.9, 10,289.9) Missing 49		0.710
Absolute lymphocyte counts (median, IQR)		4838.5 (3427.0, 5902.5) Missing 7	5217.5 (3827.7, 7334.7) Missing 49		0.710
Hb level in gm/dl (mean± SD)		9.6 ± 1.5 Missing 6	10.7 ± 1.7 Missing 47		0.086
LDH level in (U/L) (median, IQR)		516.0 (436, 1009)	477.4 (361.9, 662.1)		0.274
Anemia (missing value of CBC 53)		4 (44.4)	17 (22.4)	2.78(0.67–11.50)	0.215
Histoplasma positive in urine by PCR		1 (6.7)	7 (5.7)	1.18 (0.13–10.34)	1.000
LDH positive in blood		13 (86.7)	112 (91.1)	0.64 (0.13–3.20)	0.634
Duration of hospitalization in days (median, IQR)		10.0 (9.0, 17.0)	17.0 (12.0, 24.0)		0.128
Got both first and second line antibiotics		8 (53.3)	43 (35.0)	2.12 (0.72–6.26)	0.256
Death		7 (46.7)	11 (8.9)	8.90(2.71–29.24)	<0.001

**Table 4:** Logistic regression analysis to find out an independent risk factor for invasive fungal infection among study group children.

Variables	Adjusted OR with 95% CI	*p*-Value
Age	1.03 (0.97, 1.09)	0.306
Female sex	3.48 (1.05, 11.55)	0.042
Septic shock	4.77 (0.98, 23.22)	0.053
Danger sign of severe pneumonia	2.34 (0.68, 8.02)	0.175

## Data Availability

This data set contains some personal information of the study patients (such as name, admission date, month, area of residence). Our IRB has required that the personal information of the participants is not disclosed. Thus, the policy of our center (icddr,b) is that we should not make the availability of whole data set in the manuscript, the supplemental files, or a public repository. However, data related to this manuscript are available upon request and for researchers who meet the criteria for access to confidential data may contact with Armana Ahmed (armana@icddrb.org) to the Research Administration of icddr,b (http://www.icddrb.org/ (Last accessed on 2 January 2022).

## Supplementary Materials

The following supporting information can be downloaded at: https://www.mdpi.com/article/10.3390/life12010094/s1, Figure S1: Characteristics of children with invasive fungal infections.
