# Supplementary figures and images for: Invasive Fungal Infections in Under-Five Diarrheal Children: Experience from an Urban Diarrheal Disease Hospital
Source: Life (Basel). 2022 Jan 10;12(1):94. doi: 10.3390/life12010094 (PMC8777596; doi:10.3390/life12010094)

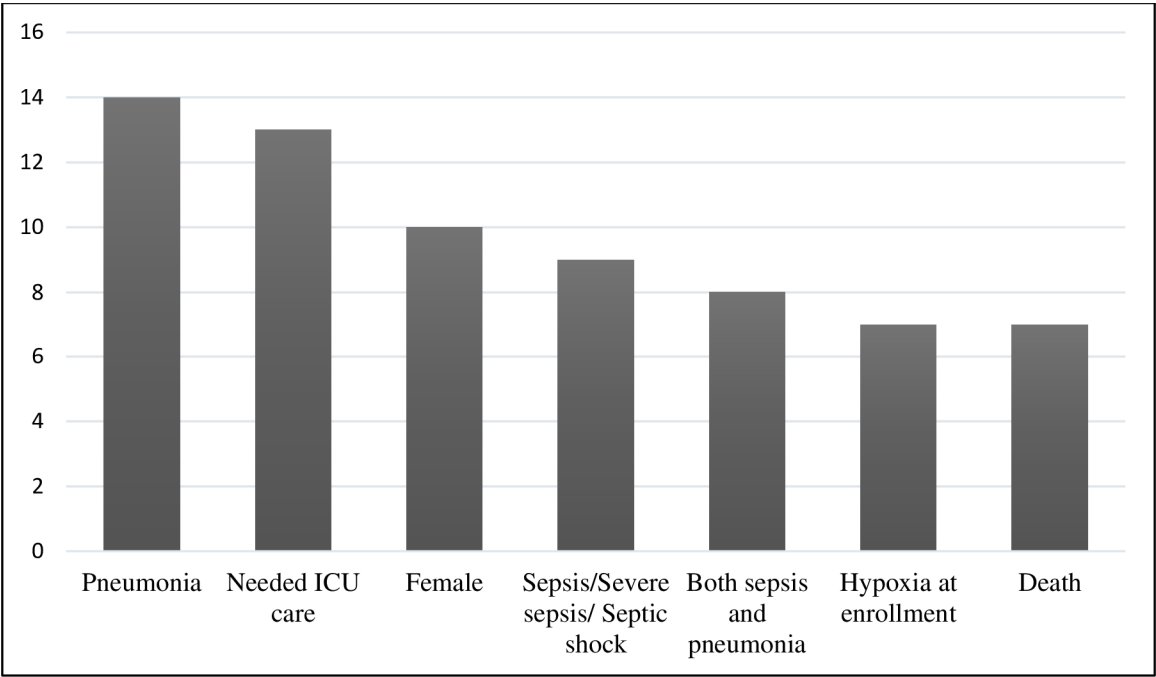

**Figure S1.** Characteristics of children with invasive fungal infections.

Supplement: Supplementary file 1 [file life-12-00094-s001.zip › life-1524168-supplementary.pdf]
